# The importance of abductor pollicis longus in wrist motions: A physiological wrist simulator study

**DOI:** 10.1016/j.jbiomech.2018.07.011

**Published:** 2018-08-22

**Authors:** Darshan S. Shah, Claire Middleton, Sabahat Gurdezi, Maxim D. Horwitz, Angela E. Kedgley

**Affiliations:** a Department of Bioengineering, Imperial College London, London, United Kingdom; b Department of Hand Surgery, Chelsea and Westminster Hospital, London, United Kingdom

**Keywords:** Wrist simulator, Muscle forces, Abductor pollicis longus

## Abstract

The abductor pollicis longus (APL) is one of the primary radial deviators of the wrist, owing to its insertion at the base of the first metacarpal and its large moment arm about the radioulnar deviation axis. Although it plays a vital role in surgical reconstructions of the wrist and hand, it is often neglected while simulating wrist motions *in vitro*. The aim of this study was to observe the effects of the absence of APL on the distribution of muscle forces during wrist motions. A validated physiological wrist simulator was used to replicate cyclic planar and complex wrist motions in cadaveric specimens by applying tensile loads to six wrist muscles – flexor carpi radialis (FCR), flexor carpi ulnaris, extensor carpi radialis longus (ECRL), extensor carpi radialis brevis, extensor carpi ulnaris (ECU) and APL. Resultant muscle forces for active wrist motions with and without actuating the APL were compared. The absence of APL resulted in higher forces in FCR and ECRL – the synergists of APL – and lower forces in ECU – the antagonist of APL. The altered distribution of wrist muscle forces observed in the absence of active APL control could significantly alter the efficacy of *in vitro* experiments conducted on wrist simulators, in particular when investigating those surgical reconstructions or rehabilitation of the wrist heavily reliant on the APL, such as treatments for basal thumb osteoarthritis.

## 1 Introduction

Of the numerous muscles in the forearm that have their tendons crossing the wrist, six muscles insert at the carpals or the base of the metacarpals – flexor carpi radialis (FCR), flexor carpi ulnaris (FCU), extensor carpi radialis longus (ECRL), extensor carpi radialis brevis (ECRB), extensor carpi ulnaris (ECU) and abductor pollicis longus (APL) – and have larger moment arms about the wrist axes (Brand and Hollister, 1999, Garland et al., 2018). Therefore, physiological wrist simulators often recreate the kinematic and kinetic conditions of the natural joint *in vitro* by applying tensile loads to tendons of these muscles (Werner et al., 1996). However, some *in vitro* studies employing wrist simulators neglect the APL, and replicate wrist motions with five actively loaded muscles (Dimitris et al., 2015, Erhart et al., 2012, Farr et al., 2013, Leonard et al., 2002).

Since *in vitro* studies using physiological simulators have direct implications for surgical reconstructions and/or rehabilitation procedures, it is important that these devices are as biofidelic as possible. In addition, the APL plays a vital role in the numerous surgical reconstructions proposed as treatments for basal thumb osteoarthritis (Avisar et al., 2015, DelSignore and Accardi, 2009, Scheker and Boland, 2004), or the reconstruction of the first dorsal interosseous (Neviaser et al., 1980) and the extensor pollicis longus (Chitnis and Evans, 1993). Consequently, the aim of this study was to observe the effects of the omission of APL on wrist biomechanics using a physiological wrist simulator. We hypothesised that the absence of APL would result in significant alterations in wrist muscle forces.

## 2 Materials and methods

### 2.1 Specimens and experimental setup

Seven fresh-frozen cadaveric specimens (five females and two males, aged 50.7 ± 9.4 years), with no history of relevant wrist disorders, were obtained from a licensed human tissue facility. Ethical approval for the use of these specimens was obtained from the Tissue Management Committee of the Imperial College Healthcare Tissue Bank, according to the Human Tissue Act. The specimens were stored at −20 °C prior to this study, and were thawed at room temperature for 12 h. The six wrist muscles considered for this study – FCR, FCU, ECRL, ECRB, ECU and APL – were dissected at their distal musculotendinous junction. All other soft tissue was resected 5 cm proximal to the wrist.

Following dissection, the specimens were mounted on a physiological wrist simulator (Shah et al., 2017). Tensile loads were applied to steel cables sutured to the distal tendons of the aforementioned wrist muscles using linear actuators (SMS Machine Automation, Barnsley, UK) mounted in-line with servo motors (Animatics Corp., Milpitas, USA). The forces applied to the tendons were monitored using load cells (Applied Measurements Ltd., UK) connected in series with the actuators. Clusters of retroreflective passive markers fixed rigidly to the third metacarpal and the radius, and anatomical landmarks recommended by the International Society of Biomechanics (Wu et al., 2005), were used to define the co-ordinate systems of the hand and the forearm, respectively. Joint angles were obtained in real-time by employing an eight-camera optical motion capture system (Qualisys, Göteborg, Sweden).

### 2.2 Simulations

Active wrist motions were replicated *in vitro* by employing a control strategy, which used position feedback to drive joint kinematics with simultaneous force feedback to ensure muscle forces remained within physiological bounds (Shah and Kedgley, 2016). The control strategy computed the distribution of forces across the wrist muscles in real-time, with iterations performed every 4–5 ms (Shah and Kedgley, 2016). The various inputs to the control strategy included specimen-specific moment arms of the tendons, determined prior to active simulations according to the passive tendon excursion method (An et al., 1983), the upper bound on muscle forces, defined as the product of muscle physiological cross-sectional area (Holzbaur et al., 2007) and specific muscle tension (Kent-Braun and Ng, 1999), and the lower bound on muscle forces, chosen according to the minimum muscle activity obtained from electromyography (Fagarasanu et al., 2004).

The control strategy was used to simulate multiple cycles of planar and complex wrist motions *in vitro*, with the hand in the vertically upward orientation. Planar wrist motions included flexion–extension (FE) – 50° flexion to 30° extension to 50° flexion (FE-5030) – and radioulnar deviation (RUD) – 15° ulnar deviation to 15° radial deviation to 15° ulnar deviation (RUD-15). Complex wrist motions included clockwise circumduction (CCD_cw_) – 30° flexion to 10° ulnar deviation to 30° extension to 10° radial deviation – and anticlockwise circumduction (CCD_acw_) – 30° flexion to 10° radial deviation to 30° extension to 10° ulnar deviation. To simulate the absence of the APL, the corresponding actuator was displaced to its maximum length, and switched off while performing active wrist motions; this ensured that no force was generated by APL during the entire range of motion.

### 2.3 Data analysis

Each specimen was moved through five cycles for all wrist motions with muscle forces evaluated at every 10° in FE and 5° in RUD, for every planar and complex wrist motion. The data were found to deviate from a normal distribution when checked for normality using the Shapiro-Wilk test (IBM SPSS Statistics, IBM Corp., Armonk, USA); hence, non-parametric tests were used to compare the data. The Wilcoxon-signed rank test was performed to observe differences in muscle forces during active motions simulated with and without the APL (significance: p < 0.05).

## 3 Results

**Fig. 1:**
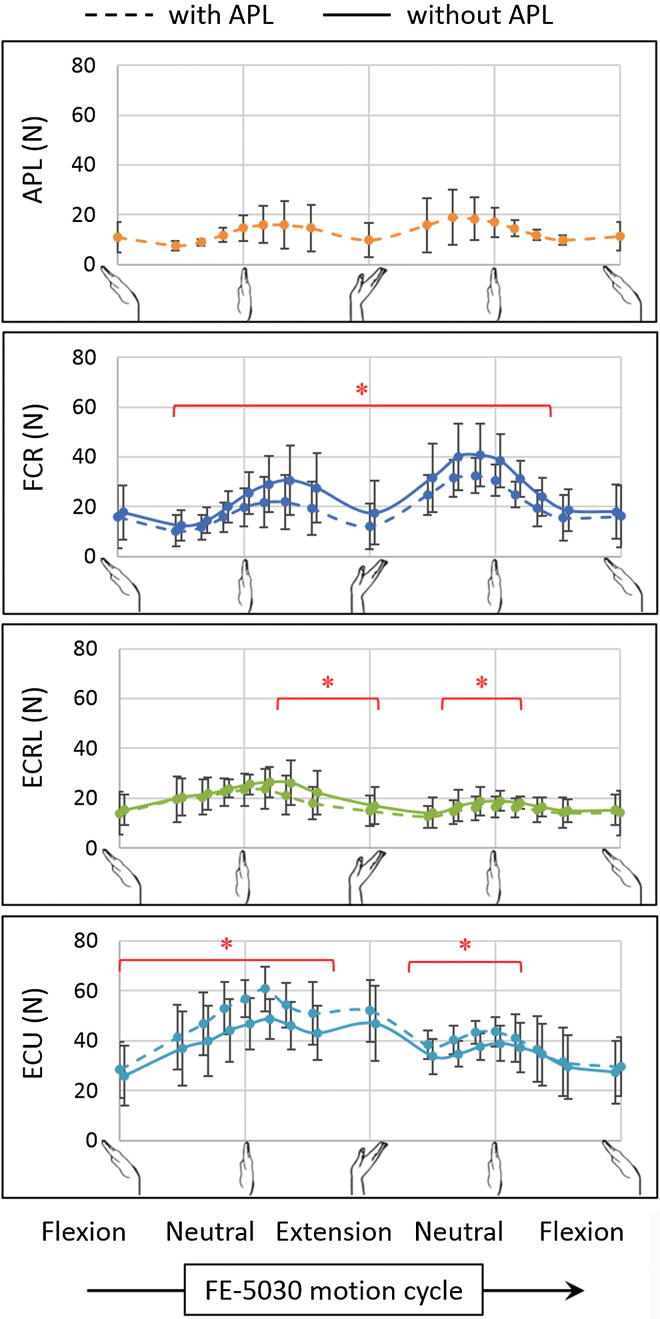
Muscle forces (mean ± one standard deviation) in flexion–extension (FE-5030) with (dashed) and without (solid) the abductor pollicis longus (APL) for flexor carpi radialis (FCR), extensor carpi radialis longus (ECRL) and extensor carpi ulnaris (ECU). The asterisk (^*^) indicates statistically significant differences between the two groups (significance: p < 0.05).

While simulating FE-5030 in the absence of the APL (Fig. 1), the mean peak FCR force increased by 21% (p = 0.018), while that of FCU, ECRB and ECU decreased by 12% (p = 0.043), 5% (p = 0.018) and 13% (p = 0.028) respectively, as compared to the muscle forces from the intact specimens. The FCR force was higher throughout the range of motion (p < 0.028), except during flexion greater than 40° (p = 0.063). Conversely, the ECU force was lower for the majority of the range of motion (p < 0.043), except at 30° extension (p = 0.063) and flexion greater than 30° (p > 0.063). The ECRL force was higher during extension greater than 10° and flexion less than 20° (p < 0.043). No difference was observed for the forces of FCU and ECRB for the majority of the range of motion, except at flexion angles between 20° and 40° for FCU (p < 0.043), and flexion angles greater than 30° for ECRB (p < 0.028).

**Fig. 2:**
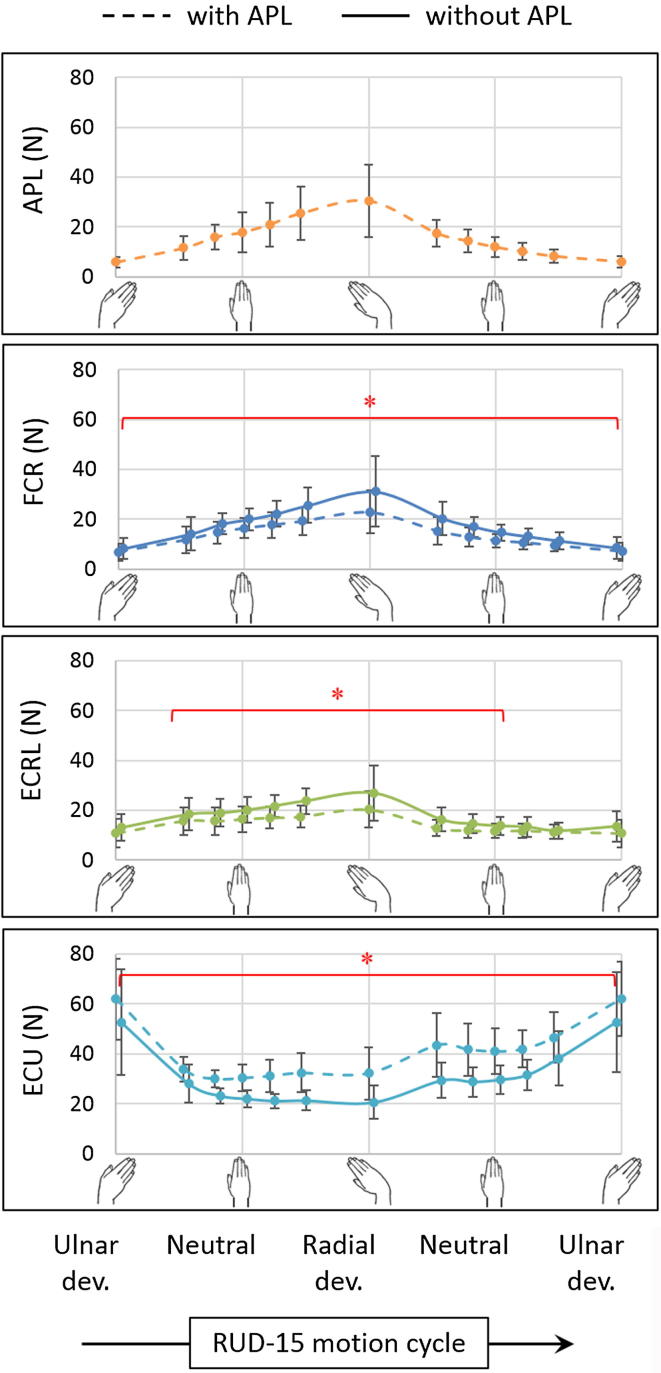
Muscle forces (mean ± one standard deviation) in radioulnar deviation (RUD-15) with (dashed) and without (solid) the abductor pollicis longus (APL) for flexor carpi radialis (FCR), extensor carpi radialis longus (ECRL) and extensor carpi ulnaris (ECU). The asterisk (^*^) indicates statistically significant differences between the two groups (significance: p < 0.05).

In the case of RUD-15 in the absence of the APL (Fig. 2), the mean peak forces of FCR and ECRL increased by 29% (p = 0.018) and 30% (p = 0.028) respectively, while that of ECU decreased by 12% (p = 0.018), when compared to the muscle forces from the intact specimens. The FCR force was higher (p < 0.028), while that of ECU was lower (p < 0.028), throughout the range of motion. The ECRL force was higher throughout radial deviation (p < 0.043). No difference was observed for the forces of FCU and ECRB throughout the range of motion (p > 0.063).

**Fig. 3:**
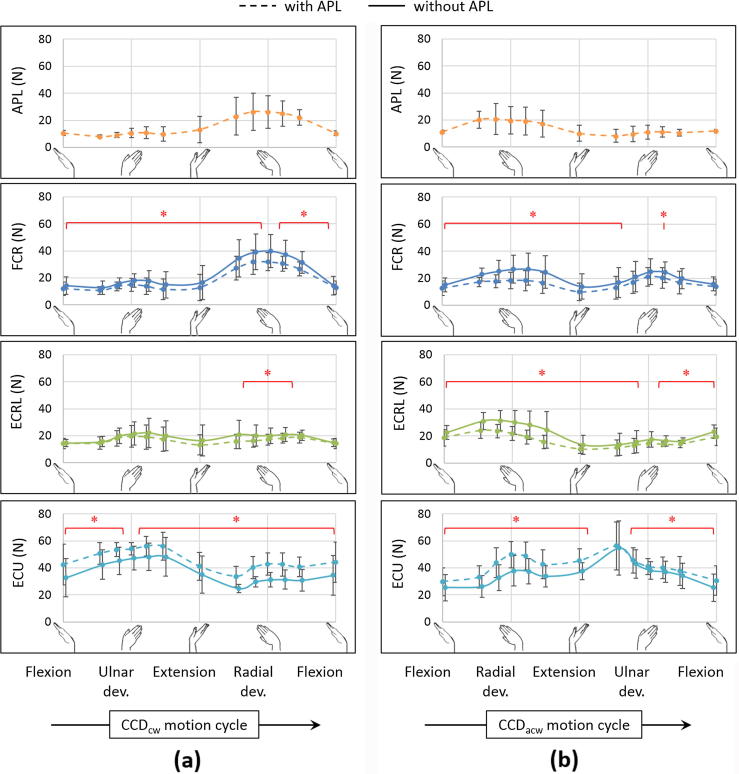
Muscle forces (mean ± one standard deviation) in (a) clockwise circumduction (CCD_cw_) and (b) anticlockwise circumduction (CCD_acw_) with (dashed) and without (solid) the abductor pollicis longus (APL) for flexor carpi radialis (FCR), extensor carpi radialis longus (ECRL) and extensor carpi ulnaris (ECU). The asterisk (^*^) indicates statistically significant differences between the two groups (significance: p < 0.05).

In the case of circumduction in the absence of the APL (Fig. 3), the mean peak FCR force increased by 24% in CCD_cw_ (p = 0.043) and 30% in CCD_acw_ (p = 0.018), the mean peak ECRL force increased by 35% in CCD_acw_ (p = 0.018), while the mean peak ECU force decreased by 10% in CCD_cw_ (p = 0.028), when compared to forces from the intact specimens. The ECU force was lower throughout the range of motion (p < 0.043), except at maximum ulnar deviation in CCD_cw_ (p = 0.063) and extension less than 20° in CCD_acw_ (p = 0.31). The FCR force was higher throughout the range of motion in CCD_cw_ (p < 0.043), except at maximum radial deviation (p = 0.063), and throughout radial deviation in CCD_acw_ (p < 0.028). The ECRL force, however, was higher during radial deviation greater than 5° in CCD_cw_ (p < 0.043), but throughout the range of motion in CCD_acw_ (p < 0.043), except at maximum ulnar deviation (p = 0.063). No difference was observed for the forces of FCU and ECRB throughout CCD_cw_ (p > 0.091), and for a majority of the range of motion in CCD_acw_ (p > 0.063), except at flexion of 10° for FCU (p = 0.028), and flexion of 20° for ECRB (p = 0.018).

## 4 Discussion

Although it is an abductor of the carpometacarpal joint of the thumb, the APL is also a strong radial deviator of the wrist, owing to its large moment arm about the radioulnar deviation axis (Garland et al., 2018). Moreover, it also acts as a stabiliser of the wrist during active FE *in vivo* (Kauer, 1980). Despite this, the APL is often neglected during the simulation of wrist motions using physiological wrist simulators (Dimitris et al., 2015, Erhart et al., 2012, Farr et al., 2013, Leonard et al., 2002). However, results from multiple cyclic planar and complex wrist motions simulated in this study, with and without the APL, showed that its absence resulted in alterations in the force distribution across the wrist muscles.

Since the APL acts as a radial flexor of the wrist (Brand and Hollister, 1999), both FCR and ECRL act as its synergists – the former as its synergist flexor, and the latter as its synergist radial deviator. Hence, in the absence of APL, the higher FCR force in both FE and RUD (Fig. 1, Fig. 2) indicated compensatory flexion torque, while the higher force of ECRL was primarily observed in radial deviation (Fig. 2). In the case of circumduction (Fig. 3), FCR compensated for the absence of APL in CCD_cw_ with higher forces throughout the range of motion, and was assisted by ECRL during radial deviation; however, the synergists switched roles in CCD_acw_, with the ECRL compensating for the absence of APL by higher forces throughout the range of motion, while being assisted by FCR in radial deviation. In contrast, since ECU acts as an antagonist of the APL about both the FE and RUD axes, the force of ECU was lower for the majority of the range for all motions. This reduced the ulnar deviation torque, thereby compensating for the reduction of radial deviation torque in the absence of APL. Lastly, the absence of APL did not alter the force profiles of FCU and ECRB, since they act as synergists of the APL about one of the wrist axes and antagonists about the other. Although statistical adjustments for multiple comparisons between muscle forces from the intact and the APL deficient group were not performed in this study, they will be considered for future studies that have direct clinical implications.

Thus, in the absence of APL, for all simulated wrist motions, the forces of FCR and ECRL were higher over portions of the range, since they act as synergists of the APL; however, the force of ECU, an antagonist ulnar extensor, was lower throughout the ranges of motion. Thus, studies performed on wrist simulators, which do not actively control the APL while replicating wrist motions *in vitro*, could potentially be overestimating the force of FCR and ECRL, while simultaneously underestimating the force of ECU. This could have significant repercussions on the reported effectiveness of surgical reconstructions being tested on such simulators, especially those heavily reliant on the APL – for instance, the reconstructive surgeries proposed as treatments for basal thumb osteoarthritis, wherein the APL is employed to create load-bearing support slings to avoid the collapse of the first metacarpal in the absence of a trapezium (DelSignore and Accardi, 2009, Scheker and Boland, 2004), or to ensure the placement of a prosthesis and prevent implant loosening (Avisar et al., 2015).

This study reports findings from wrist motions replicated *in vitro* on a validated physiological wrist simulator (Shah et al., 2017) using a control strategy previously shown to have low kinematic error and high repeatability (Shah and Kedgley, 2016, Shah et al., 2017). In addition, the differences in muscle forces observed with and without the APL were greater than the repeatability of muscle forces on the simulator (Shah et al., 2017), which confirmed that the results were reflective of the absence of APL. One of the main advantages of the control strategy was the use of specimen-specific muscle moment arms about the wrist axes as custom inputs. This accounted for the variations in APL observed across specimens, due to the presence of multiple distal tendon slips (Thwin et al., 2014), which is one of the possible reasons for the omission of the APL during *in vitro* testing on other simulators reported in the literature (Dimitris et al., 2015, Erhart et al., 2012, Farr et al., 2013, Leonard et al., 2002). However, the variations of the APL moment arm about the FE and adduction-abduction axes of the carpometacarpal joint (Smutz et al., 1998) were not considered in this study, since the position of the first metacarpal was not actively controlled. Another limitation of the simulator was the use of six muscles to simulate wrist motions in the cadaveric specimens, since these muscles primarily affect the wrist. The extrinsic muscles of the finger and thumb were not loaded while simulating wrist motions because they have smaller moment arms about the wrist axes (Brand and Hollister, 1999), and their principal effect is distal to the wrist; moreover, their inclusion would require a significantly more complex control strategy. However, these muscles will be considered in future experiments, not only because tendons of all muscles passing through the wrist contribute to the overall wrist torque, but also since certain extrinsic muscles have been known to contribute significantly to wrist stability *in vivo* (Kauer, 1980). The inclusion of extrinsic muscles of the thumb would further facilitate the active control of the carpometacarpal joint, thereby enabling the inclusion of APL moment arm variations about the carpometacarpal joint and quantification of the effects of thumb kinematics on muscle forces.

In conclusion, even though the APL is primarily an abductor of the carpometacarpal joint of the thumb, its effects on the wrist should not be overlooked during *in vitro* simulations or surgical reconstruction.
